# Three new species of *Camptoscaphiella* Caporiacco, 1934 (Araneae, Oonopidae) from Yunnan Province, China

**DOI:** 10.3897/BDJ.11.e109679

**Published:** 2023-09-08

**Authors:** Xiaohan Wang, Zengxue Wang, Yanfeng Tong, Dongju Bian, Zizhong Yang

**Affiliations:** 1 Life Science College, Shenyang Normal University, Shenyang 110034, China Life Science College, Shenyang Normal University Shenyang 110034 China; 2 Key Laboratory of Forest Ecology and Management, Institute of Applied Ecology, Chinese Academy of Sciences, Shenyang 110016, China Key Laboratory of Forest Ecology and Management, Institute of Applied Ecology, Chinese Academy of Sciences Shenyang 110016 China; 3 National-Local Joint Engineering Research Center of Entomoceutics, Dali University, Yunnan Dali, 671000, China National-Local Joint Engineering Research Center of Entomoceutics, Dali University Yunnan Dali, 671000 China

**Keywords:** Asia, biodiversity, goblin spiders, new taxa, taxonomy

## Abstract

**Background:**

*Camptoscaphiella* Caporiacco, 1934 is a small genus of oonopid spiders that currently contains 20 species, of which five have been recorded in Yunnan, China.

**New information:**

Three new species of *Camptoscaphiella*, *C.hudie* Tong & Yang, **sp. nov.** (female), *C.yinglefeng* Tong & Yang, **sp. nov.** (female, male) and *C.yujufeng* Tong & Yang, **sp. nov.** (male) are described from Yunnan, China. Descriptions, diagnoses and photographs are provided.

## Introduction

Oonopidae is a diverse spider family with 1932 extant described species in 115 genera. The genus *Camptoscaphiella* Caporiacco, 1934 is mainly distributed in tropical and subtropical montane regions of Asia, mostly within the Himalayan Plateau. Only two species have been recorded in the Paciﬁc island of New Caledonia (Baehr and Harvey 2013, Grismado et al. 2014, World Spider Catalog 2023). Members of the genus are tiny and typically have remarkable morphology of the male palps, which have an extremely large, club-shaped palpal patella and a bulb that is well separated from the cymbium and the first two pairs of legs, which have extremely long spines with the tibiae bearing four pairs of spines and the metatarsi bearing two pairs of spines (Baehr and Ubick 2010).

All five species of *Camptoscaphiella* known from China are currently recorded in Yunnan Province, i.e. *C.changxu* Tong & Li, 2021, *C.linyejiei* Tong & Li, 2021, *C.paquini* Ubick, 2010, *C.sinensis* Deeleman-Reinhold, 1995 and *C.tuberans* Tong & Li, 2007 (Deeleman-Reinhold 1995, Tong and Li 2007, Baehr and Ubick 2010, Huang et al. 2021).

In this paper, three new *Camptoscaphiella* species, *C.hudie* sp. nov., *C.yinglefeng* sp. nov. and *C.yujufeng* sp. nov., collected from Cangshan Mountain, Yunnan Province, are described and illustrated.

## Materials and methods

The specimens used in this study were collected by pitfall trapping and later examined using a Leica M205C stereomicroscope. Details of body parts and copulatory organs were studied under an Olympus BX51 compound microscope. Endogynes were cleared in lactic acid and left male palps were removed to provide detailed illustrations. Photos were made with a Canon EOS 750D zoom digital camera (18 megapixels) mounted on an Olympus BX51 compound microscope. Scanning electron microscope images (SEM) were taken under high vacuum with a Hitachi S-4800, specimens were air-dried and sputter-coated using IXRF SYSTEMS. All measurements were taken using an Olympus BX51 compound microscope and are given in millimetres.

Type material is deposited in Shenyang Normal University (SYNU) in Liaoning, China.

The following abbreviations are used in the text and figures: ALE = anterior lateral eyes; ap = apodemes; as = anterior sclerite; cd = copulatory duct; PLE = posterior lateral eyes; PME = posterior median eyes; psr = posterior scutal ridge; rlf = retrolateral fold; spr = semicircular, prolateral rim; tmp = triangular median plate; tss = triangular sclerotised structure; va = ventral appendices; vp = ventral process.

## Taxon treatments

### Camptoscaphiella hudie

Tong & Yang sp. nov.

6DD015E1-FB24-5F53-B5F7-5ACED40D9077

4BB6BDF4-09AB-4364-BC98-0CFEACB97393

#### Materials

- **Type status:** Holotype. **Occurrence:** recordedBy: Rong Huang & Depeng Xu; individualID: SYNU-670; individualCount: 1; sex: female; lifeStage: adult; preparations: whole animal; occurrenceID: 8325A7FC-AB10-5A60-AE6B-5DFE1A6C48C3; **Taxon:** order: Araneae; family: Oonopidae; genus: Camptoscaphiella; specificEpithet: *hudie*; scientificNameAuthorship: Tong & Yang; **Location:** country: China; stateProvince: Yunnan; county: Dali City; locality: Cangshan Mountain, post-fire forest in 2008; verbatimCoordinates: 25°38′52″N, 100°07′15″E; **Identification:** identifiedBy: Yanfeng Tong; **Event:** eventDate: 15 November 2008

#### Description

**Figure 1.**
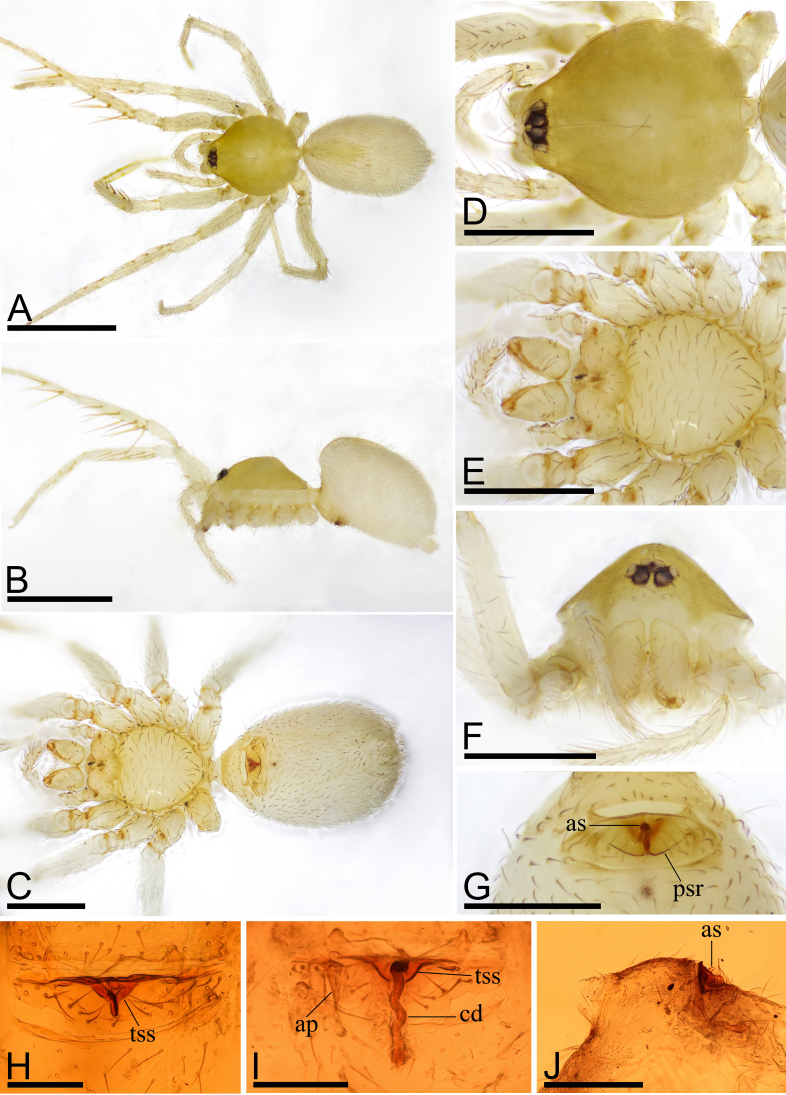
*Camptoscaphiellahudie* **sp. nov**., holotype female. **A** habitus, dorsal view; **B** habitus, lateral view; **C** habitus, ventral view; **D** prosoma, dorsal view; **E** prosoma, ventral view; **F** prosoma, anterior view; **G** epigastric region, ventral view; **H** epigastric region, ventral view; **I** endogyne, dorsal view; **J** epigastric region, lateral view. Abbreviations: ap = apodemes, as = anterior sclerite, cd = copulatory duct, psr = posterior scutal ridge, tss = triangular sclerotised structure. Scales: A, B = 0.8 mm; C–F = 0.4 mm; G = 0.2 mm; H–J = 0.1 mm.

Female (Holotype). Body: pale yellow, abdomen and legs yellowish-white; habitus as in Fig. 1A–C; length 1.64. Carapace (Fig. 1D and F): 0.70 long, 0.59 wide; pars cephalica strongly elevated in lateral view, surface of elevated portion and sides of pars cephalica finely reticulate. Eyes (Fig. 1D and F): ALE 0.052; PME 0.042; PLE 0.039; posterior eye row procurved from both above and front; ALE separated by less than radius. Clypeus (Fig. 1F): margin unmodified, straight in front view, sloping forward in lateral view. Mouthparts (Fig. 1E and F): chelicerae unmodified; endites distally not excavated, serrula present in single row. Sternum (Fig. 1E): as long as wide, surface finely reticulate, with small inter-coxal, triangular extensions for coxae III and IV. Abdomen (Fig. 1A–C): 0.94 long, 0.60 wide; dorsal scutum very small and narrow, covering about 1⁄2 of abdomen length, 1⁄6 of abdomen width, not fused to epigastric scutum; postepigastric scutum small, widely hexagonal, only around epigastric furrow. Legs (Fig. 1A and B): femur I additionally with 1 long prolateral spine. Epigastric area (Fig. 1G, H and J): with small triangular anterior sclerite (as), situated in middle of epigastric area; with pair of wing-shaped posterior scutal ridge (psr). Endogyne (Fig. 1I): with anterior triangular sclerotised structure (tss); copulatory duct (cd) long and narrow with slightly broadened tip reaching beyond posterior groove; apodemes (ap) short.

Male: unknown.

#### Diagnosis

The new species is similar to *Camptoscaphiellapanchthar* Baehr, 2010, but can be distinguished by the very small dorsal scutum of abdomen (Fig. 1A) vs. about 1/3 of abdomen width (Baehr and Ubick (2010): fig. 291) and the wing-shaped posterior scutal ridge of epigastric region (Fig. 1G) vs. lacking the scutal ridge, having instead a large pear-shaped median plate (Baehr and Ubick (2010): figs. 297, 298).

#### Etymology

The specific epithet is derived from Chinese pinyin, “hudie”, which means “butterfly”, referring to the wing-shaped posterior scutal ridge; noun in apposition.

#### Distribution

Known only from the type locality.

### Camptoscaphiella yinglefeng

Tong & Yang sp. nov.

D12BA89A-10FE-5B76-B780-9F6E5B5A8CA2

2A3BF2A2-26BD-4DFE-BC05-D0D6B3D15FB0

#### Materials

- **Type status:** Holotype. **Occurrence:** recordedBy: Zizhong Yang; individualID: SYNU-693; individualCount: 1; sex: male; lifeStage: adult; preparations: whole animal; occurrenceID: 50F43FDD-9358-5A58-8E23-57DE4C5CDD76; **Taxon:** order: Araneae; family: Oonopidae; genus: Camptoscaphiella; specificEpithet: *yinglefeng*; scientificNameAuthorship: Tong & Yang; **Location:** country: China; stateProvince: Yunnan; county: Dali City; locality: Cangshan Mountain,Yinglefeng Hill; verbatimCoordinates: 25°41′28″N, 100°5′48″E; **Identification:** identifiedBy: Yanfeng Tong; **Event:** eventDate: 8 Febuary 2010
- **Type status:** Paratype. **Occurrence:** recordedBy: Zizhong Yang; individualID: SYNU-694-699; individualCount: 6; sex: 4 females, 2 males; lifeStage: adult; preparations: whole animal; occurrenceID: E391CDDC-0CF8-53DF-AB21-77E80C58519A; **Taxon:** order: Araneae; family: Oonopidae; genus: Camptoscaphiella; specificEpithet: *yinglefeng*; scientificNameAuthorship: Tong & Yang; **Location:** country: China; stateProvince: Yunnan; county: Dali City; locality: Cangshan Mountain, Yinglefeng Hill; verbatimCoordinates: 25°41′28″N, 100°5′48″E; **Identification:** identifiedBy: Yanfeng Tong; **Event:** eventDate: 8 Febuary 2010
- **Type status:** Paratype. **Occurrence:** recordedBy: Jianchun Zhang & Guanxu Ma; individualID: SYNU-682-692; individualCount: 11; sex: 8 females 3 males; lifeStage: adult; preparations: whole animal; occurrenceID: 9B44CA6F-EE7A-5193-8C15-6E5B32288F54; **Taxon:** order: Araneae; family: Oonopidae; genus: Camptoscaphiella; specificEpithet: *yinglefeng*; scientificNameAuthorship: Tong & Yang; **Location:** country: China; stateProvince: Yunnan; county: Dali City; locality: Cangshan Mountain,Yujufeng Hill; verbatimCoordinates: 25°41′45″N, 100°6′32″E; **Identification:** identifiedBy: Yanfeng Tong; **Event:** eventDate: 9 August 2011
- **Type status:** Paratype. **Occurrence:** recordedBy: Jianchun Zhang & Guanxu Ma; individualID: SYNU-680-681; individualCount: 2; sex: 2 females; lifeStage: adult; preparations: whole animal; occurrenceID: 6C050DF5-A0FB-5ABC-A759-E30B9A9F2B63; **Taxon:** order: Araneae; family: Oonopidae; genus: Camptoscaphiella; specificEpithet: *yinglefeng*; scientificNameAuthorship: Tong & Yang; **Location:** country: China; stateProvince: Yunnan; county: Dali City; locality: Cangshan Mountain, Dapojing; verbatimCoordinates: 225°34′28″N, 100°8′49″E; **Identification:** identifiedBy: Yanfeng Tong; **Event:** eventDate: 29 November 2008
- **Type status:** Paratype. **Occurrence:** recordedBy: Ping Feng; individualID: SYNU-679; individualCount: 1; sex: female; lifeStage: adult; preparations: whole animal; occurrenceID: 66DDC4C2-5108-5C03-B751-4E311058D9D7; **Taxon:** order: Araneae; family: Oonopidae; genus: Camptoscaphiella; specificEpithet: *yinglefeng*; scientificNameAuthorship: Tong & Yang; **Location:** country: China; stateProvince: Yunnan; county: Dali City; locality: Cangshan Mountain, post-fire forest in 2008; verbatimCoordinates: 25°38′52″N, 100°07′15″E; **Identification:** identifiedBy: Yanfeng Tong; **Event:** eventDate: 20 August 2008
- **Type status:** Paratype. **Occurrence:** recordedBy: Zhenxing Yang & Youliang Zhang; individualID: SYNU-678; individualCount: 1; sex: male; lifeStage: adult; preparations: whole animal; occurrenceID: 09F7A51F-356E-5180-8338-094B8B44C485; **Taxon:** order: Araneae; family: Oonopidae; genus: Camptoscaphiella; specificEpithet: *yinglefeng*; scientificNameAuthorship: Tong & Yang; **Location:** country: China; stateProvince: Yunnan; county: Dali City; locality: Cangshan Mountain, post-fire forest in 2008; verbatimCoordinates: 25°38′52″N, 100°07′15″E; **Identification:** identifiedBy: Yanfeng Tong; **Event:** eventDate: 12 October 2008

#### Description

**Figure 2.**
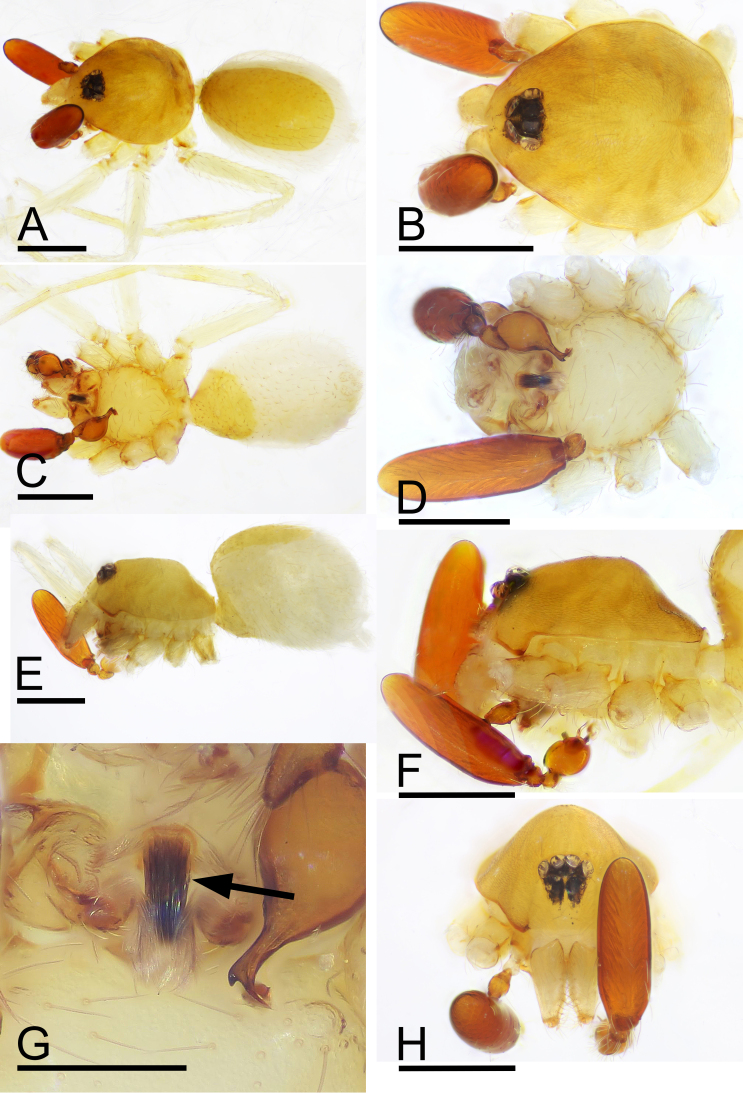
*Camptoscaphiellayinglefeng* sp. nov., male (SYNU-693). **A** habitus, dorsal view; **B** prosoma, dorsal view; **C** habitus, ventral view; **D** prosoma, ventral view; **E** habitus, lateral view; **F** prosoma, lateral view; **G** labium and endites, ventral view, arrow shows the cluster of strong setae; **H** prosoma, anterior view. Scales: A–F, H = 0.4 mm; G = 0.2 mm .

**Figure 3.**
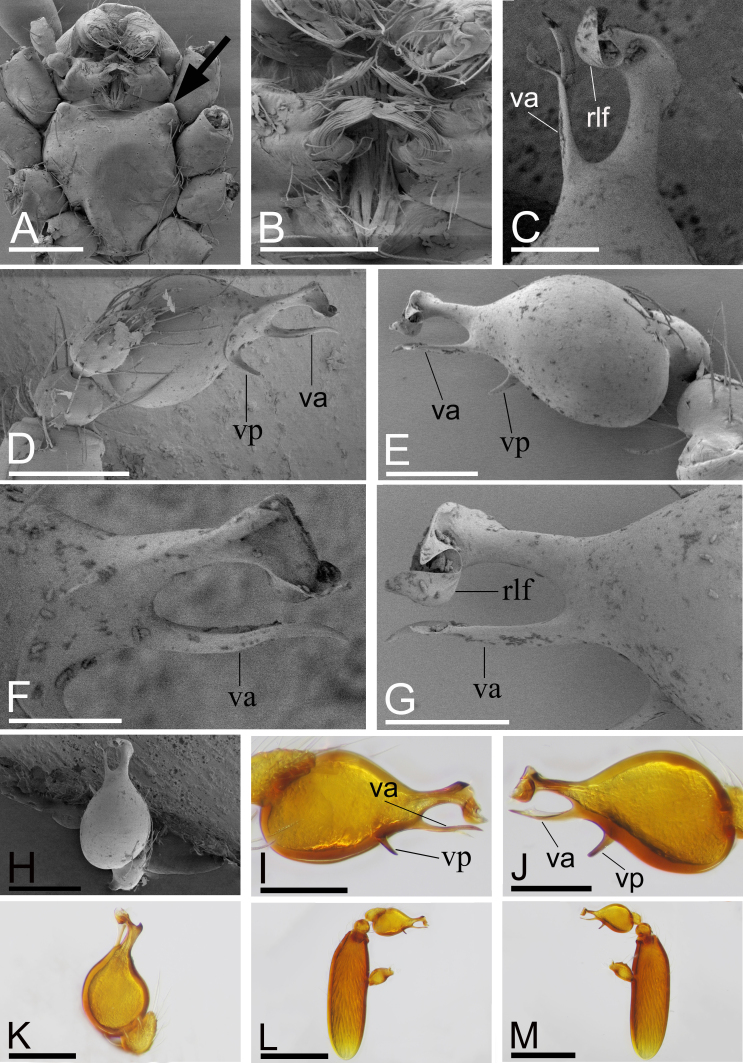
*Camptoscaphiellayinglefeng* **sp. nov.**, male (SYNU-699), A–H (SEM) microphotographs and I–M (light). **A** prosoma, ventral view, arrow shows the anterolateral bumps; **B** labium and endites, ventral view; **C** distal part of bulb, dorsal view; **D** left bulb, prolateral view; **E** left bulb, retrolateral view; **F** distal part of bulb, prolateral view; **G** distal part of bulb, retrolateral view; **H** left bulb, dorsal view; **I** left bulb, prolateral view; **J** left bulb, retrolateral view; **K** left bulb, dorsal view; **L** left palp, prolateral view; **M** left palp, retrolateral view. Abbreviations: rlf = retrolateral fold, va = ventral appendices, vp = ventral process. Scales: A, L, M = 0.2 mm; B, D, E, H–K = 0.1 mm; C, F, G = 0.05 mm.

Male (Holotype). Body: yellow, abdomen ventrally and laterally paler, whitish; habitus as in Fig. 2A, C and E; length 1.65. Carapace (Fig. 2B and F): 0.76 long, 0.63 wide; pars cephalica slightly elevated in lateral view, surface of elevated portion and sides of pars cephalica finely reticulate. Eyes (Fig. 2B,H): ALE 0.078; PME 0.064; PLE 0.062; posterior eye row procurved from both above and front; ALE separated by less than radius. Clypeus (Fig. 2B, H): margin unmodified, straight in front view, sloping forward in lateral view. Mouthparts (Fig. 2D, G and H, Fig. 3A and B): chelicerae unmodified; labium with a cluster of black, strong setae; endites with characteristic brush-like long hairs. Sternum (Fig. 2D and Fig. 3A): as long as wide, with pointed anterolateral bumps, with small inter-coxal, triangular extensions for coxae III and IV. Abdomen (Fig. 2A, C and E): 0.89 long, 0.75 wide; oval; dorsal scutum covering about 5⁄6 of abdomen length, 2⁄3 of abdomen width, anteriorly fused to epigastric scutum; postepigastric scutum small, just near epigastric furrow. Legs: yellowish-white. Palp (Fig. 3C–M): reddish-brown; patella extremely long club-shaped, length/width = 3.04, ca. 5 times the femur length and 2.4 times the bulb length; cymbium narrow in dorsal view; bulb ventrally with short and sharp spine-shaped process (vp), sub-distally with long, bifid appendices (va) and apically with retrolateral fold (rlf).

**Figure 4.**
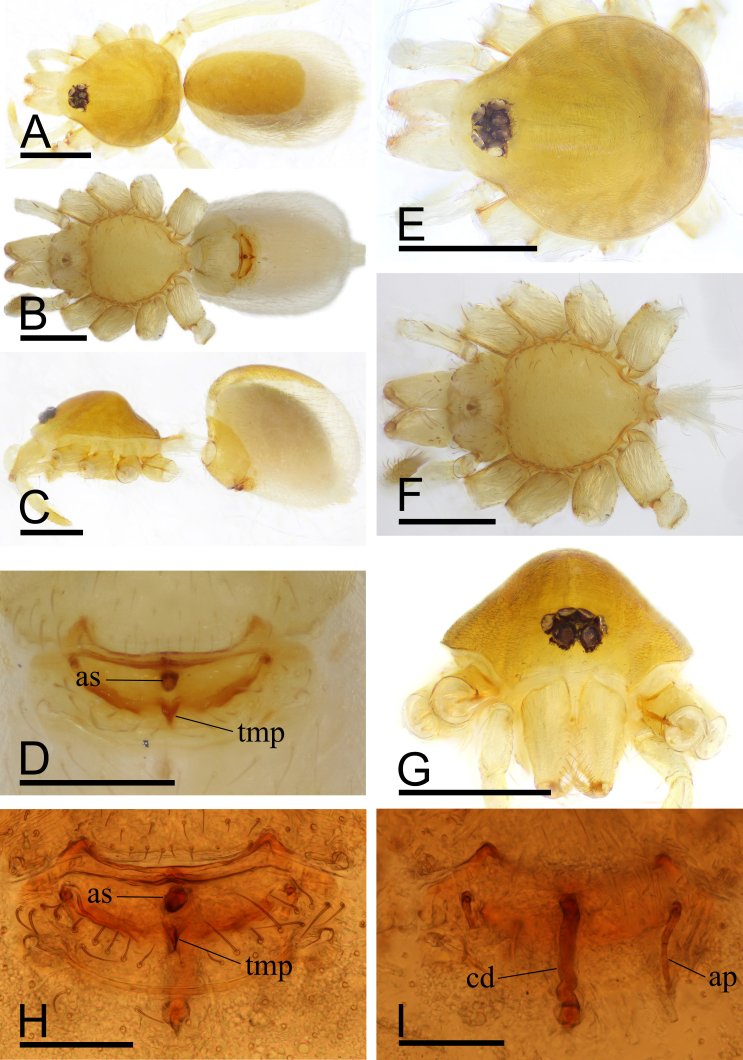
*Camptoscaphiellayinglefeng* **sp. nov.**, female (SYNU-694). **A** habitus, dorsal view; **B** habitus, ventral view; **C** habitus, lateral view; **D** epigastric region, ventral view; **E** prosoma, dorsal view; **F** prosoma, ventral view; **G** prosoma, anterior view; **H** epigastric region, ventral view; **I** endogyne, dorsal view. Abbreviations: ap = apodemes, as = anterior sclerite, cd = copulatory duct, tmp = triangular median plate. Scales: A–C, E–G = 0.4 mm; D = 0.2 mm; H–I = 0.1 mm.

Female (SYNU-694). Body: habitus as in Fig. 4A–C; length 1.76. Carapace: 0.72 long, 0.66 wide. Eyes: ALE 0.061; PME 0.053; PLE 0.046. Abdomen: 1.04 long, 0.70 wide. Epigastric area (Fig. 4D, H): with rounded anterior sclerite (as) and triangular median plate (tmp). Endogyne (Fig. 4I): copulatory duct (cd) long, narrow, straight with tip reaching far beyond posterior groove; apodemes (ap) slender.

#### Diagnosis

This new species is similar to *Camptoscaphiellatuberans* Tong & Li, 2007, but can be distinguished by the cluster of black, strong setae on the labium (Fig. 2G) vs. absent (Tong and Li (2007): fig. 23), the ventral process (vp) on subdistal part of bulb (Fig. 3I, J) vs. absent (Tong and Li (2007): fig. 25), by lacking the semicircular, prolateral rim on bulb distal part (Fig. 3I, J) vs. present (Tong and Li (2007): fig. 25) and the triangular median plate of epigastric region (Fig. 4D) vs. absent (Tong and Li (2007): fig. 22).

#### Etymology

The specific epithet is derived from the type locality; noun in apposition.

#### Distribution

Known only from the type locality.

### Camptoscaphiella yujufeng

Tong & Yang sp. nov.

CD3C2398-9763-58F5-8342-374AD5279B99

06C045B5-C3C4-4338-A064-32ABF89C2853

#### Materials

- **Type status:** Holotype. **Occurrence:** individualID: SYNU-677; individualCount: 1; sex: male; lifeStage: adult; preparations: whole animal; occurrenceID: DFE28A62-85E8-5213-8FE5-F3506856321D; **Taxon:** scientificName: *Camptoscaphiellayujufeng*; order: Araneae; family: Oonopidae; genus: Camptoscaphiella; scientificNameAuthorship: Tong & Yang; **Location:** country: China; stateProvince: Yunnan; county: Dali City; locality: Cangshan Mountain,Yujufeng Hill; verbatimCoordinates: 25°41′45″N, 100°6′32″E; **Identification:** identifiedBy: Yanfeng Tong; **Event:** eventDate: 9 August 2011

#### Description

**Figure 5.**
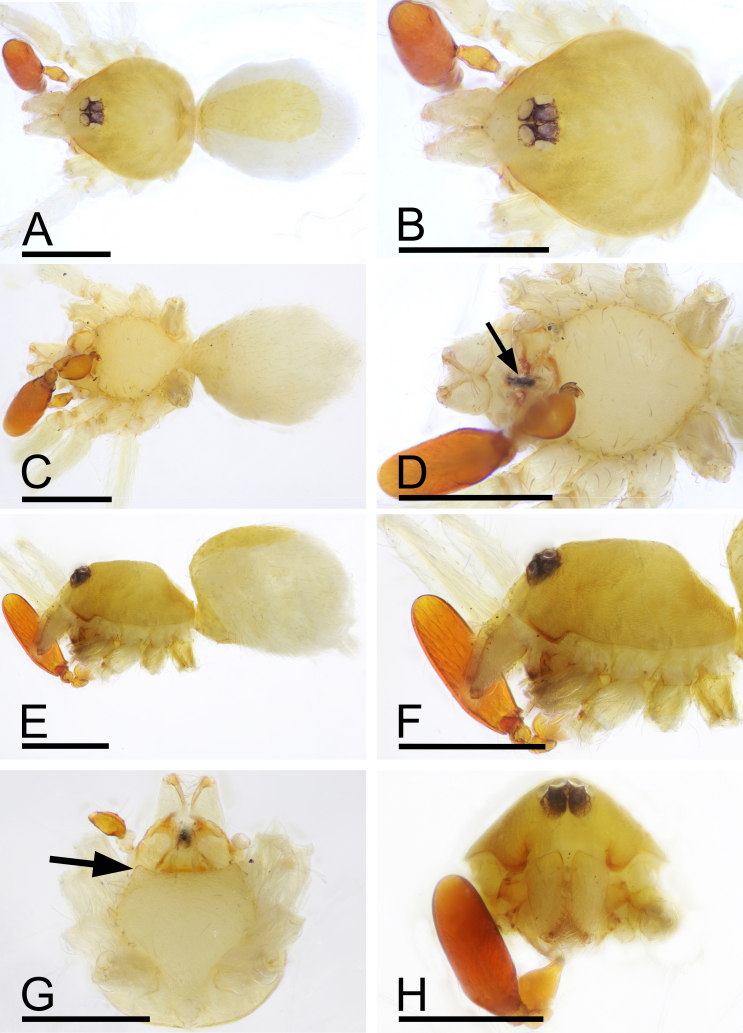
*Camptoscaphiellayujufeng* sp. nov., male. **A** habitus, dorsal view; **B** prosoma, dorsal view; **C** habitus, ventral view; **D** prosoma, ventral view, arrow shows the cluster of strong setae; **E** habitus, lateral view; **F** prosoma, lateral view; **G** prosoma, ventral view, arrow shows the anterolateral bumps; **H** prosoma, anterior view. Scales: 0.4 mm.

**Figure 6.**
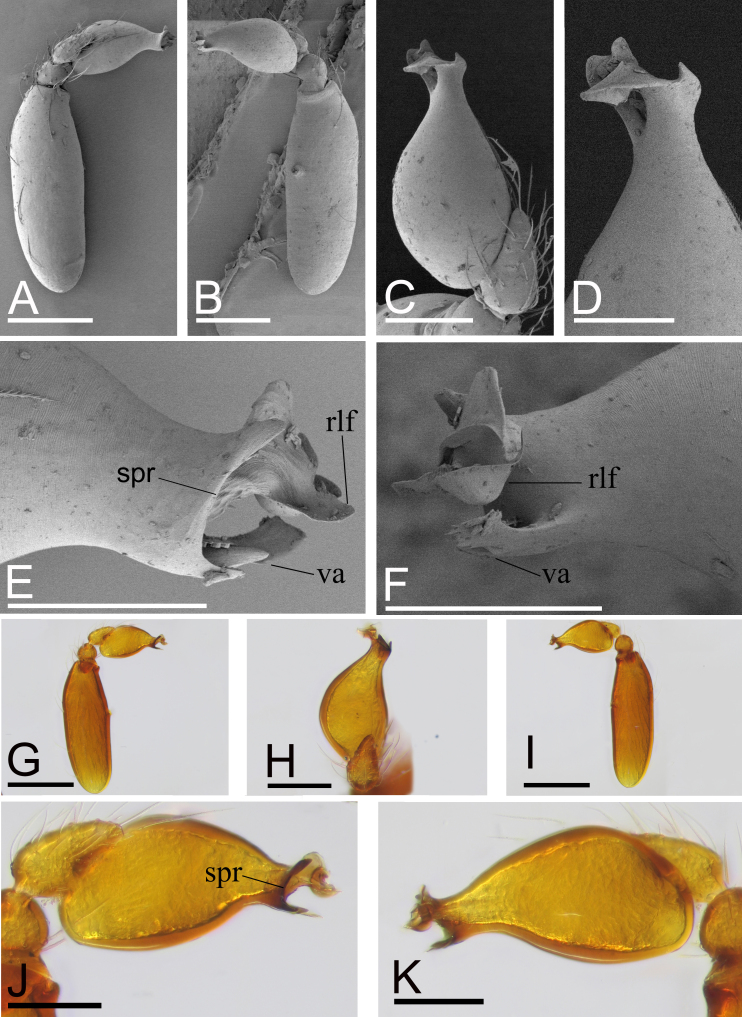
*Camptoscaphiellayujufeng* sp. nov., right palp (images flipped horizontally), A–F (SEM) microphotographs and G–K (light). **A** left palp, prolateral view; **B** left palp, retrolateral view; **C** palpal bulb, dorsal view; **D** distal part of bulb, dorsal view; **E** distal part of bulb, prolateral view; **F** distal part of bulb, retrolateral view; **G** left palp, prolateral view; **H** palpal bulb, dorsal view; **I** left palp, retrolateral view; **J** palpal bulb, prolateral view; **K** palpal bulb, retrolateral view. Abbreviations: rlf = retrolateral fold, spr = semicircular, prolateral rim, va = ventral appendices. Scales: A, B, G, I = 0.2 mm; C, H, J, K = 0.1 mm; D, E, F = 0.05 mm.

Male (Holotype). Body: pale yellow, abdomen paler; habitus as in Fig. 5A, C and E; length 1.37. Carapace (Fig. 5B and F): 0.64 long, 0.56 wide; pars cephalica slightly elevated in lateral view, surface of elevated portion and sides of pars cephalica finely reticulate. Eyes (Fig. 5B and H): ALE 0.057; PME 0.049; PLE 0.049; posterior eye row procurved from both above and front; ALE separated by less than one radius. Clypeus (Fig. 5B, F and H): margin unmodified, straight in front view, sloping forward in lateral view. Mouthparts (Fig. 5G and H): chelicerae unmodified; with a cluster of black, strong setae on the labium. Sternum (Fig. 5G): as long as wide, surface finely reticulate, with pointed anterolateral bumps. Abdomen (Fig. 5A, C and E): 0.73 long, 0.58 wide; dorsal scutum covering ca. 2⁄3 of abdomen length, 1/3 of abdomen width, anteriorly fused to epigastric scutum; postepigastric scutum small, just near epigastric furrow. Legs: yellowish-white. Palp (Fig. 6A–K): reddish-brown; patella extremely long club-shaped, length/width = 2.76, ca. 3.7 times the femur length and 2.1 times the bulb length; cymbium narrow in dorsal view; bulb distal part with semicircular, prolateral rim (spr), three-forked ventral appendices (va) and distally with small retrolateral fold (rlf) and several outgrowths.

Female: unknown.

#### Diagnosis

This new species is similar to *Camptoscaphiellayinglefeng* sp. nov., but can be distinguished by the dorsal scutum ca. 1/3 of abdomen width (Fig. 5A), vs. 2⁄3 of abdomen width (Fig. 2A), the ventral appendices of bulb shorter than retrolateral fold (Fig. 6E and F), vs. longer than retrolateral fold (Fig. 3F and G) and lacking the ventral process (Fig. 6J and K) of bulb, vs. present (Fig. 3I and J).

#### Etymology

The specific epithet is derived from the type locality; noun in apposition.

#### Distribution

Known only from the type locality.

#### Comment

*Camptoscaphiellahudie* sp. nov. (male unknown) and *Camptoscaphiellayujufeng* sp. nov. (female unknown) were collected from the same locality, Cangshan Mountain. The dorsal abdominal scutum of *C.hudie* is very small and narrow, quite different from that of *C.yujufeng* (compare Fig. 1A and Fig. 5A). This suggests that they are most likely different species.

## Supplementary Material

XML Treatment for Camptoscaphiella hudie

XML Treatment for Camptoscaphiella yinglefeng

XML Treatment for Camptoscaphiella yujufeng
